# Editorial: Targeting pumps, channels and transporters for the treatments of vascular, cardiovascular and kidney diseases

**DOI:** 10.3389/fphar.2023.1130882

**Published:** 2023-02-01

**Authors:** Jinwei Zhang, Keith Siew, Dandan Sun

**Affiliations:** ^1^ Institute of Cardiovascular Diseases, Xiamen Cardiovascular Hospital Xiamen University, School of Medicine, Xiamen University, Xiamen, Fujian, China; ^2^ State Key Laboratory of Bioorganic & Natural Products Chemistry, Center for Excellence in Molecular Synthesis, Shanghai Institute of Organic Chemistry, Chinese Academy of Sciences, Shanghai, China; ^3^ Hatherly Laboratories, Institute of Biomedical and Clinical Sciences, Medical School, Faculty of Health and Life Sciences, University of Exeter, Exeter, United Kingdom; ^4^ Department of Renal Medicine, University College London, London, United Kingdom; ^5^ Department of Neurology, University of Pittsburgh, Pittsburgh, PA, United States; ^6^ Veterans Affairs Pittsburgh Healthcare System, Geriatric Research, Education, and Clinical Center, Pittsburgh, PA, United States

**Keywords:** cardiovascular disease, vascular disease, kidney disease, ion channel, ion transporter, signal transduction, therapeutics

Pumps, channels, and transporters are essential transmembrane proteins of living cells. They transport of ions or small molecules across the cellular membrane for the normal physiological function of cardiovascular, kidney, and nervous systems. For example, the electrical impulses that travel along nerve cells to muscle cells ultimately stimulate muscle contraction triggered by the opening and closing of voltage-gated ion channels in the plasma membrane of these cells. The signaling molecule for muscle contraction and relaxation is, in fact, Ca^2+^, whose concentration in the cytoplasm of muscle cells is co-ordinately modulated by Ca^2+^-dependent channels, pumps, and transporters. Moreover, the osmotic homeostasis across the membrane is maintained with the transport of Cl^−^, Na^+^ and K^+^ ions, along with obligated H_2_O, across the plasma membrane of all animal cells by the channels, pumps and transporters. Whereas impaired ion transport regulation may cause pathophysiological changes of cellular function and cell volume dysregulation.

Proper maintenance of ion channels, transporters, and pumps is critical in a range of cellular activities, such as signal transduction, muscle contraction, volume regulation, growth, motility, apoptosis, as well as the vascular ion and water homeostasis. Dysregulation of ion channels, transporters or pumps can result in numerous vascular diseases including stroke, peripheral artery disease, abdominal aortic aneurysm, carotid artery disease, arteriovenous malformation, critical limb-threatening ischemia, pulmonary embolism (blood clots), deep vein thrombosis, chronic venous insufficiency, and varicose veins; cardiovascular diseases including coronary heart disease, cerebrovascular disease, peripheral arterial disease, rheumatic heart disease, congenital heart disease, deep vein thrombosis, and pulmonary embolism; and kidney diseases including acute and chronic kidney disease, diabetes, fluid and electrolyte disorders, glomerulonephritis and glomerular disease, lupus, hypertension, kidney and pancreas transplantation, and kidney-related metabolic disorders. The Human Genome Project has identified more than 406 putative ion channels and 533 extracellular transporter proteins (Venter et al., 2001); however only a limited number of them has been explored. From those Food and Drug Administration (FDA)-approved drugs that on 667 identified unique human protein efficacy targets and 189 identified pathogen protein efficacy targets, approximately 18%–19% of these targets are ion channels (Santos et al., 2017), highlighting the importance of ion channels as drug targets in the treatment of disease.

The Research Topic includes three original research papers and two reviews from prominent researchers in the field and provides readers of the journal with recent results or summaries in the area of mechanisms of ion channels and transporters in vascular, cardiovascular and kidney diseases, as well as new strategies for treatment or drug screenings.

The ether-a-go-go (EAG) channels are a family of voltage-gated K^+^ channels. One member of the family, the human *ether-á-go-go-related* gene (hERG) K^+^ channel, is crucial for the repolarization of the cardiac ventricular action potential, as it conducts a rapidly delayed rectifier K^+^ current (*I*_Kr_) (Abbott et al., 1999). Increased susceptibility to arrhythmias has been found in pathological cardiac hypertrophy (pCH) with a reduction of *I*_Kr_ (Rahm et al., 2018). However, practical approaches to prevent *I*_Kr_ deficiency have long been sought. Zhang et al., found that the factors responsible for hERG channel remodelling were Nedd4-2 activation by hypertrophy and that Nedd4-2-dependent ubiquitination was critically involved in *I*_Kr_ deficiency in a guinea pig model of cardiac hypertrophy induced by Angiotensin II (Ang II). They showed that overexpression of the inactive form of Nedd4-2 was able to reverse *I*_Kr_ reduction, reverse long QT, and decrease arrhythmias. Furthermore, a newly synthesized PY motif containing peptide was shown to rescue the myocardial remodelling by restoring the *I*_Kr_ deficiency, with a reduction of Nedd4-2. This discovery improves our understanding of hERG channel remodelling, and could pave the new way for novel anti-arrhythmia therapy.

Transmembrane protein 16A (TMEM16A, also known as anoctamin 1, ANO1) was identified as a Ca^2+^ -activated chloride channel (CaCC) (Caputo et al., 2008; Schroeder et al., 2008; Yang et al., 2008), expressed in smooth muscle and many other tissues. It is known to contribute to controlling vascular endothelial, smoothing muscle tone, and regulating cardiac myocyte excitability (Shang et al., 2020), or regulating the ClCa channel conductance and the proliferation of portal vein smooth muscle cells (PVSMCs) in portal hypertension (Zeng et al., 2018). Kondo et al. investigated the transcriptional and functional change of TMEM16A in cirrhotic and non-cirrhotic portal hypertension animal models and found the expression of the TMEM16A was downregulated in PVSMCs of the former model, leading to reduced ClCa currents and increased spontaneous contractions of portal veins. Such higher amplitude frequency of spontaneous contractions could be counteracted by the administration of a specific inhibitor of TMEM16A channels, T16A_inh_-A01. This study suggests that TMEM16A ClCa channels are involved in the pathological mechanisms underlying cirrhotic portal hypertension and could be used as novel drug targets for therapeutic interventions.

Transient Receptor Potential Melastatin 3 (TRPM3), a Ca^2+^ permeable non-selective cation channel, has been found to be expressed in kidney cells and is important for renal Ca^2+^ homeostasis (Grimm et al., 2003), as well as in sensory nerve cells, where it acts as a heat sensor (Vriens et al., 2011). Liu et al. comprehensively analyzed the expression and clinical features of calcium-related genes in kidney renal clear cell carcinoma (KIRC) patients based on the Cancer Genome Atlas (TCGA) database. The authors found that the calcium-related genes were correlated with T cell-related immune pathways such as T cell differentiation, T cell-mediated immunity, T cell cytokine production, T cell proliferation and migration, and T cell receptor signaling pathway, through calculating the Clinical Risk Groups (CRGs)-related risk score. Along with experimental validation, the study found TRPM3 mRNA and protein expression were significantly lower in KIRC patients than in normal controls, and lower TRPM3 expression was associated with poor prognosis in KIRC patients. The study suggests that immunotherapy combined with a modulation of calcium signaling through TRPM3 may be a promising strategy for the treatment of KIRC.

Hydrogen sulfide (H_2_S) is an important gas transmitter and has been shown to play a vital role in many physiological and pathological processes within the vascular systems. Liu et al. reviewed and discussed the role of H_2_S in the regulation of vascular tone, especially through interaction with different vascular potassium channels and the underlying mechanisms. The authors concluded that some diseases, including hypertension, are associated with decreased synthesis of endogenous H_2_S, which could be a potential therapeutic target for the treatment of vascular diseases.

The transient receptor potential (TRP) multigene superfamily encodes integral membrane proteins that function as ion channels, for examples, TRPV1, Piezo1 and Piezo2 ion channels, allow unparalleled flexibility and maintain intracellular Ca^2+^ homeostasis, playing important roles in many physiological functions, such as sensing pressure or temperature in our bodies (Zhang et al., 2022). Fallah et al. provided an overview regarding to TRP ion channels, the role of TRP channels in health and disease conditions, TRP channels as potential drug targets, drugs targeting on TRP ion channels, and ion channel screening technologies. In particular, the authors highlighted the application of Aurora’s Ion Channel Reader (ICR), an updated, radioactive-free version of the ion flux assay platform, for studying ion channels and high-throughput drug screening.

Overall, this Research Topic summarizes important findings and recent research progress related to ion channels and transporters, their mechanisms of regulation, and new strategies of treatment for vascular, cardiovascular and kidney diseases. The Research Topic provides new research findings about the roles of ion channels and transporters in disease pathogenesis and their potential as therapeutic targets.
